# High-Performance Photodetectors Based on Semiconducting Graphene Nanoribbons

**DOI:** 10.1021/acs.nanolett.3c03563

**Published:** 2023-11-27

**Authors:** Mingyang Wang, Xiaoxiao Zheng, Xiaoling Ye, Wencheng Liu, Baoqing Zhang, Zihao Zhang, Rongli Zhai, Yafei Ning, Hu Li, Aimin Song

**Affiliations:** † Shandong Technology Centre of Nanodevices and Integration, School of Microelectronics, Shandong University, Jinan 250101, China; ‡ Shenzhen Research Institute of Shandong University, Shenzhen 518063, China; § Department of Electrical and Electronic Engineering, University of Manchester, M13 9PL Manchester, U.K.

**Keywords:** Graphene nanoribbon, photodetector, heterojunction, responsivity

## Abstract

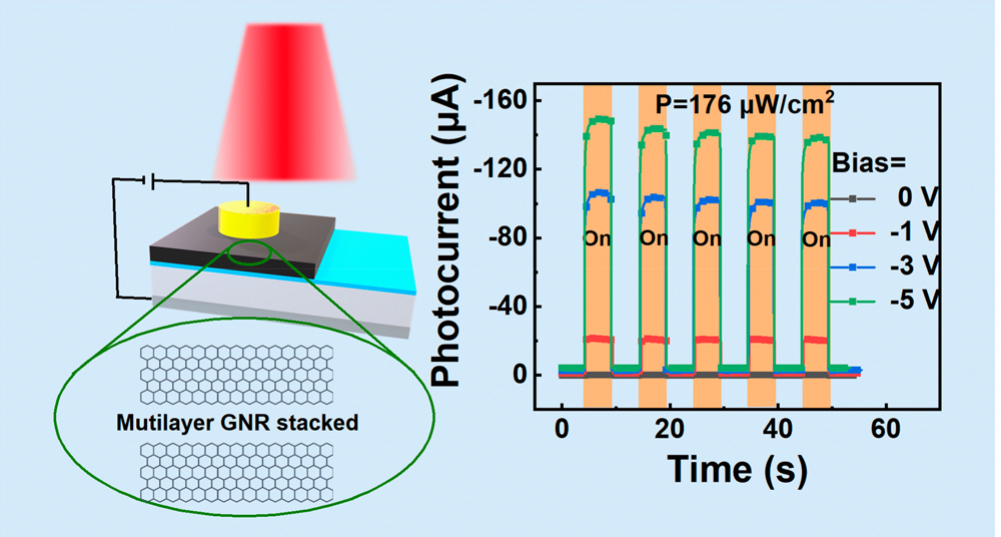

The inherent zero-band gap nature of graphene and its fast photocarrier recombination rate result in poor optical gain and responsivity when graphene is used as the light absorption medium in photodetectors. Here, semiconducting graphene nanoribbons with a direct bandgap of 1.8 eV are synthesized and employed to construct a vertical heterojunction photodetector. At a bias voltage of −5 V, the photodetector exhibits a responsivity of 1052 A/W, outperforming previous graphene-based heterojunction photodetectors by several orders of magnitude. The achieved detectivity of 3.13 × 10^13^ Jones and response time of 310 μs are also among the best values for graphene-based heterojunction photodetectors reported until date. Furthermore, even under zero bias, the photodetector demonstrates a high responsivity and detectivity of 1.04 A/W and 2.45 × 10^12^ Jones, respectively. The work shows a great potential of graphene nanoribbon-based photodetection technology.

The past decade has witnessed a tremendous development of graphene in a variety of fields ranging from flexible electronics, energy storage, gas detections to medical diagnosis.^1−10^ Owing to the unique gapless electronic structure, graphene can absorb photons of a very wide wavelength range (from ultraviolet to terahertz), and it is therefore considered as a promising material for photodetectors.^11,12^ However, graphene-based photodetectors unfortunately suffer from low responsivity in practical devices due to the inherent fast photocarrier recombination rate and the low optical absorption efficiency.^6^ The lifetime of the photoexcited carriers in graphene is on the order of a few picoseconds, and one must separate the electron–hole pairs within a time scale that is less than the lifetime of the photocarrier in order to generate a considerable photocurrent. Consequently, most reported graphene-based photodetectors, e.g. phototransistors and photoconductive detectors, where graphene is used as a light absorption medium, exhibit weak optical responsivity commonly below hundreds of mA/W.^13−15^

One strategy to enhance the performance of graphene-based photodetectors is by integrating graphene with semiconductors such as Si,^12^ MoS_2_^16^ and ZnO^17^ to form heterojunctions, in which graphene performs as a transparent Schottky electrode to collect carriers instead of a light absorption medium. Among them, graphene-Si heterojunction photodetectors show unique superiorities due to the compatibility of graphene preparation technology with silicon processing technology, rendering it a potential for large-scale integration into photodetector networks.^12^ Kulandaivel et al. reported a self-powered photodetector based on a graphene-Si Schottky junction which exhibited a high responsivity of 510 mA/W and a photo switching ratio of 10^5^ under the illumination of a 532 nm light source.^18^ Chen et al. reported a graphene-Si short-wave infrared photodetector. The synergistic effects of plasmonics and electron trapping in their device made it exhibit an ultrahigh responsivity of 83 A/W.^19^ An et al. reported a graphene-Si heterojunction photodetector with a tunable photoresponsivity up to 435 mA/W under 850 nm irradiation.^20^ Zhu et al. demonstrated a vertical structure reduced graphene oxide-Si photodetector with a maximum responsivity of 63 mA/W.^21^ Additionally, owing to the high dark currents caused by the high conductivity of graphene, most graphene-based photodetectors exhibit low detectivity usually below to 10^13^ Jones.^12,20,21^ The optoelectrical performance of graphene photodetectors still has great potential in light of their responsivity and detectivity. Therefore, it is highly desirable to achieve a graphene-based heterojunction photodetector with significantly enhanced optoelectrical performance by enhancing the absorption efficiency of graphene or improving the lifetime of the photogenerated carriers.

Here, in order to overcome the low optical absorption and responsivity caused by the zero-bandgap of graphene, p-type semiconducting graphene nanoribbons (GNR) with a bandgap of 1.81 eV and n-type Si were employed to fabricate a heterojunction photodetector. A thin layer of Al_2_O_3_ is inserted between the GNRs and Si to serve as both a blocking layer and a passivation layer. This layer increases the interfacial barrier and passivates defects to reduce the leakage current transport path, thus suppressing the dark current, thereby improving the properties of photodetectors. The results demonstrate that the semiconducting GNR-based photodetector shows an ultrahigh responsivity of 1052 A/W, outperforming previous graphene-based heterojunction photodetectors by 1–4 orders of magnitude. A high detectivity of 3.13 × 10^13^ Jones and a fast response time of 310 μs are achieved, which are also among the best values for reported graphene-based heterojunction photodetectors. The photodetector can also operate in a self-powered mode and exhibit a responsivity and detectivity of 1.04 A/W and 2.45 × 10^12^ Jones. These results indicate that semiconducting GNR-based photodetectors hold great potential in future high-performance and low-cost photodetectors.

The process flow of GNR-based heterojunction photodetectors is presented in Figure 1a. The GNR fabrication methods and device fabrication methods can be found in the Supporting Information. GNRs are obtained by unzipping the single-walled carbon nanotubes (SWCNTs), the reaction mechanism can be found in our previous work.^22^ Atomic force microscopy (AFM) imaging is used to confirm the successful formation of GNRs by unzipping SWCNTs. Figure S1a,b shows the AFM images of a pristine SWCNT and a GNR on a Si/SiO_2_ substrate, respectively. The crossing-height distribution reveals a height of 1.24 nm of a SWCNT and a height of 0.53 nm of a GNR, respectively, indicating a successful unzipping of SWCNTs.^22^ The cross-sectional image of the fabricated GNR/Al_2_O_3_/Si heterojunction device is shown in Figure 1b. Figure 1c shows the Raman spectra of pristine SWCNTs and fabricated GNRs, respectively. A strong G peak located at the Raman shift of 1590 cm^–1^ and a weak D peak located at the Raman shift of 1343 cm^–1^ are observed in the Raman spectrum of pristine SWCNTs. After SWCNTs are unzipped to form GNRs, a stronger D peak is observed in the Raman spectrum, which is attributed to the GNR edges and defects induced by the process of unzipping SWCNTs.^23^ To further confirm the successful formation of GNRs with an opening bandgap, the photoluminescence characterization is applied on the pristine SWCTNs and GNRs. As shown in Figure 1d, a sharp photoluminescence peak is observed at a wavelength of 686 nm, suggesting a bandgap of 1.81 eV of GNRs. In contrast, no photoluminescence peak is observed in pristine SWCNTs, further proving the successful formation of GNRs by unzipping SWCNTs. Notably, the PL spectrum of GNRs exhibits a narrow full width at half-maximum of below to 10 nm. It is well-known that the bandgap of a GNR is inherently contingent upon its width. The breadth of the PL spectrum peak is directly related to the homogeneity of the bandgap distribution within the GNRs. Hence, the utilization of SWCNTs with uniform diameters and an optimized preparation process serves as the pivotal factor behind the attainment of GNRs with consistent widths and correspondingly uniform bandgap distributions. This is the main reason for such a narrow peak of PL spectrum of GNRs. In addition, the low level of defects and the flat edges in GNRs may also be beneficial to form a narrow PL peak.

**Figure 1:**
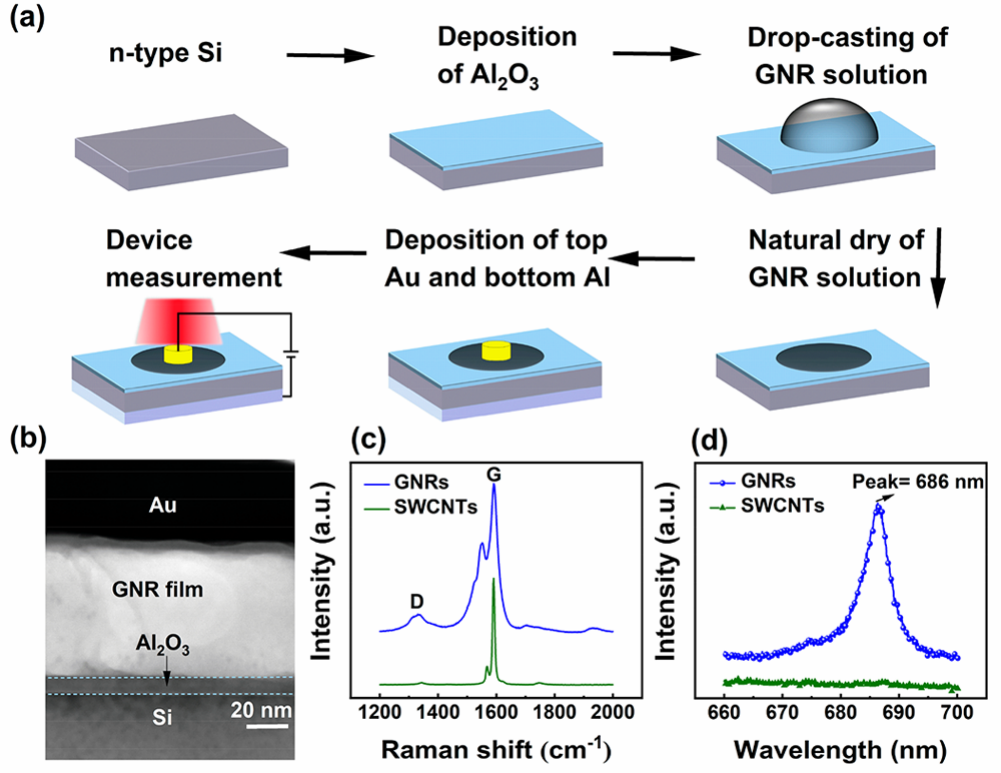
(a) Process flow of GNR/Al_2_O_3_/Si photodetectors. (b) Cross-sectional image of the fabricated device. (c) Raman spectra of pristine SWCNTs and fabricated GNRs. (d) Photoluminescence spectra of SWCNTs and GNRs.

The fundamental feature of a p-n heterojunction is its rectifying characteristic. As shown in Figure 2a, the current–voltage (*I*–*V*) curve of the GNR/Si heterojunction without the Al_2_O_3_ interfacial layer measured under dark conditions reveals a high reverse saturation current of ∼0.8 mA and a forward saturation current of ∼0.08 mA. The heterojunction exhibits no rectifying behavior within the applied bias voltage range of −5 to +5 V. The possible reason is that SWCNTs are not fully unzipped and the residual highly conductive metallic SWCNTs in GNR film lead to a large reverse current. In contrast, as shown in Figure 2b, the reverse saturation current of the GNR/Si heterojunction with the Al_2_O_3_ interfacial layer is reduced to 3.6 μA under dark conditions, over 2 orders of magnitude lower than that of the device without the Al_2_O_3_ interfacial layer. This is mainly attributed to the effective passivation of the heterojunction interface traps by the Al_2_O_3_ layer, which reduces the generation of holes and electrons in the depletion region under reverse bias voltage.^24^ The forward current (∼0.04 mA) in the GNR/Al_2_O_3_/n-Si heterojunction is slightly reduced due to the energy barrier effects of Al_2_O_3_ on the carrier transportation but comparable to the GNR/Si heterojunction (∼0.08 mA). This demonstrates the effective tunneling of carriers while passing through the Al_2_O_3_ interfacial layer.^25^ Due to the presence of an Al_2_O_3_ interfacial layer, the GNR/Al_2_O_3_/n-Si heterojunction exhibits a typical rectifying behavior with a rectifying ratio of over 10.

**Figure 2:**
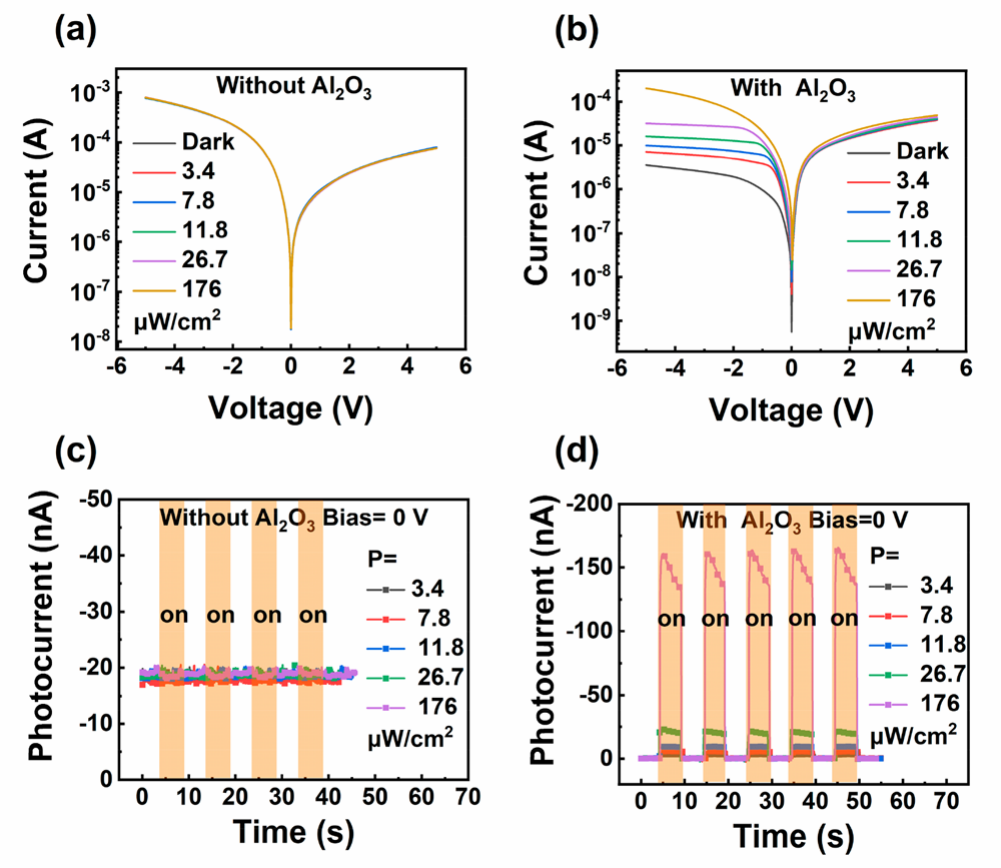
Current–voltage (*I*–*V*) curves of GNR/Si photodetectors without (a) and with (b) the Al_2_O_3_ interfacial layer measured under 635 nm laser irradiation at a light power density varying from 3.4 to 176 μW/cm^2^. Dynamic current response of GNR/Si photodetectors without (c) and with (d) an Al_2_O_3_ interfacial layer at 0 V bias.

In order to investigate the effect of the Al_2_O_3_ interfacial layer on the photodetection performance of the devices, the *I*–*V* curves of vertically stacked GNR/Si and GNR/Al_2_O_3_/Si devices were measured under dark and 635 nm laser illumination with a light power density varying from 3.4 μW/cm^2^ to 176 μW/cm^2^, where the voltage between the top gold electrode and the bottom aluminum electrode varies from −5 to +5 V. As shown in Figure 2a, *I*–*V* curves of the GNR/Si device measured under dark conditions completely overlap with those measured under laser illumination. This phenomenon reveals that the GNR/Si device without an Al_2_O_3_ interfacial layer exhibits a poor photodetection ability, which is attributed to the high dark current at the reverse voltage region. In comparison to the magnitude of the dark current, the minute photocurrent generated by photogenerated carriers under laser illumination exhibits negligible influence on the overall device current. In contrast, Figure 2b shows the *I*–*V* curves of the GNR/Al_2_O_3_/Si device measured under dark conditions and laser illumination with different light power densities. Both the reverse current and forward current under 635 nm laser illumination are obviously higher than those under dark conditions, indicating a significant enhancement of the photodetection performance of the GNR/Si heterojunction with an Al_2_O_3_ interfacial layer. As shown in Figure 2c, no obvious photocurrents were generated when the laser was constantly turned on and off. In contrast, the GNR/Al_2_O_3_/Si device exhibits substantial photocurrents when the laser is turned on, the magnitude of which is proportional to the incident laser power, as shown in Figure 2d. When the bias voltage is 0 V, a high photocurrent of 166 nA is observed under a light power density of 176 μW/cm^2^, demonstrating the potential for self-powered photodetectors. In the GNR/Al_2_O_3_/Si heterojunction device, when a thermal equilibrium state is formed, the depletion region will generate a built-in electric field directed from Si to GNR which is induced by the carrier diffusion process. The built-in electric field will drive the separation of photogenerated electron–hole pairs. Thus, when the laser is on, the photogenerated electrons will transfer from GNR to Si, the photogenerated holes will transfer from Si to GNR due to the built-in electric field, leading to a reverse photocurrent under 0 V bias. To elucidate the origin of such a remarkable photoresponse, we measured the *I*–*V* curves of Au/Al_2_O_3_/Si device without GNR layer under a laser irradiation with a power of 176 uW/cm^2^ and the dynamic photocurrent response at different bias voltages. Figure S3 illustrates that, under laser illumination, both reverse and forward currents of the Au/Al_2_O_3_/Si device exhibited no discernible increase compared with dark conditions. The device did not generate continuous observable photocurrents at different bias voltages; instead, it solely displayed transient current disturbances coinciding with the laser’s activation and deactivation. This finding demonstrates that the remarkable photoresponse observed in the GNR/Al_2_O_3_/Si photodetector primarily stems from the optical absorption of the graphene nanoribbon layer.

The separation process of photogenerated hole–electron pairs under laser illumination can be influenced by the applied reverse bias voltage. Figure 3a exhibits the dynamic current response of the GNR/Al_2_O_3_/Si device measured at a light power density of 176 μW/cm^2^ under 0, −1, −3 and −5 V bias. The photocurrents increase with increasing reverse bias and exhibit rapid response times under all bias voltages. In Figure 3a, the photocurrent indeed decreases gradually at a bias voltage of −5 V. It is not uncommon in photodetectors that the photocurrent exhibits reduction during the beginning period time of testing under a reverse bias.^26^ This instability may be related to some of the carriers gaining a high energy and subsequently injecting into some high trapping states at the interface or inside the Al_2_O_3_ barrier. As these trap states become saturated and become inactive with time, the photocurrent eventually stabilizes at a constant value as shown in Figure S6. Figure 3b shows the photocurrent of the device at different light power densities as a function of the applied reverse bias voltages. Under 176 μW/cm^2^ laser illumination, as the bias voltage changes from 0 to −5 V, the photocurrent of the device increases from 0.17 to 196 μA, indicating a 3 orders of magnitude increase. The dynamic current response of the device at a bias voltage of −5 V under different light power densities is shown in Figure 3c. Compared to the photocurrents at 0 V bias (Figure 2d), the photocurrents at all light power densities are greatly enhanced at a −5 V bias, indicating that the photocurrents can be tunable by applying bias voltages. The photodark current ratio (PDCR) is defined as^27^
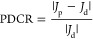
1where the *J*_p_ and *J*_d_ are the photocurrent density and the dark current density. The PDCRs of the GNR/Al_2_O_3_/Si device at 0 and −5 V bias as a function of the light power density are shown in Figure 3d. The highest PDCR of 1538 is obtained at a bias voltage of 0 V and a light power density of 176 μW/cm^2^. However, the highest PDCR decreases to 37 when the bias voltage is −5 V, which is attributed to the high dark current at the bias voltage of −5 V (3.6 μA). Figure 3e and 3f demonstrate the light power density dependence of the device photocurrent under bias voltages of 0 and −5 V, respectively. The linear fitting results indicate a better linearity (with an outstanding value of *R*^2^ = 0.9998) under 0 V bias, suggesting a reliable light power density can be inferred from the observed photocurrent.

**Figure 3:**
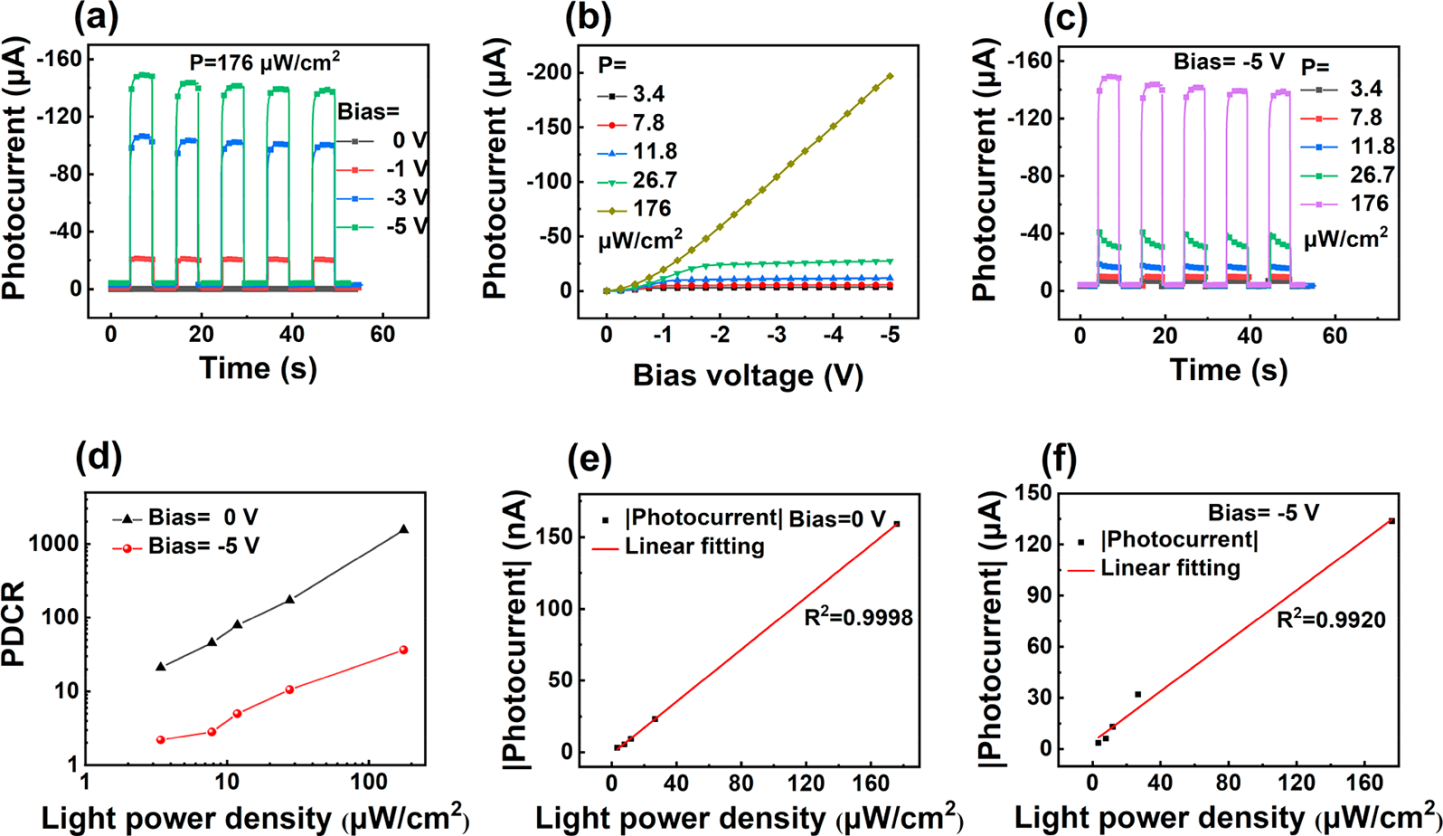
(a) Photocurrents of the GNR/Al_2_O_3_/Si photodetector as a function of bias voltages measured at different light power densities. (b) Dynamic current response measured under bias voltages of 0, −1, −3, −5 V, respectively. (c) Dynamic current response measured at the light power density of 3.4, 7.8, 11.8, 26.7, and 176 μW/cm^2^, respectively. (d) PDCR as a function of the light power density under the bias voltage of 0 and −5 V. The dependence of photocurrents measured at bias voltages of 0 V (e) and −5 V (f) with light power density.

To further evaluate the photodetection performance of the GNR/Al_2_O_3_/Si device, the photo responsivity (*R*), detectivity (D*) and external quantum efficiency (EQE) are extracted from following equations:^28−30^
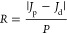
2
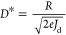
3
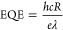
4where *P*, *e*, *h*, *c* and λ are the light power density, elementary charge, Planck constant, velocity of light in vacuum and the wavelength of laser irradiation, respectively. In general, the responsivity and the external quantum efficiency of photodetectors are used to evaluate its sensitivity. The detectivity indicates the ability of a photodetector to recognize weak optical signals. The dependence of photocurrent, *R*, D* and EQE on the bias voltage at a light power density of 3.4 μW/cm^2^ are shown in Figure 4a, b and c, respectively. The highest *R*, D* and EQE are 1052 A/W, 3.13 × 10^13^ Jones and 2 × 10^5^ % obtained at the bias voltage of −5 V. To the best of our knowledge, the responsivity is the highest value compared to reported graphene-based heterojunction photodetectors. In pn heterojunction-based photodetectors, photogenerated electron–hole pairs can be efficiently separated by an internal built-in voltage. Separated charges with opposite polarities will induce opposite Coulomb potentials to balance the built-in potentials in the junctions. Thus, the transport current through the heterojunction will increase, leading to a higher EQE.^31^ Moreover, when the device is operated under reverse bias, the EQE is further increased because the larger reverse voltage further reduces the injection barrier and enhances the electric field inside the junction making the photogenerated current increase dramatically.^32^ To evaluate the response speed of the device, an oscilloscope is used to measure the photovoltage variation within a single laser pulse, as shown in Figure 4d. The rise time (τ_r_) and decay time (τ_d_) are extracted to be 5.6 ms and 310 μs, indicating a fast photoresponse speed.

**Figure 4:**
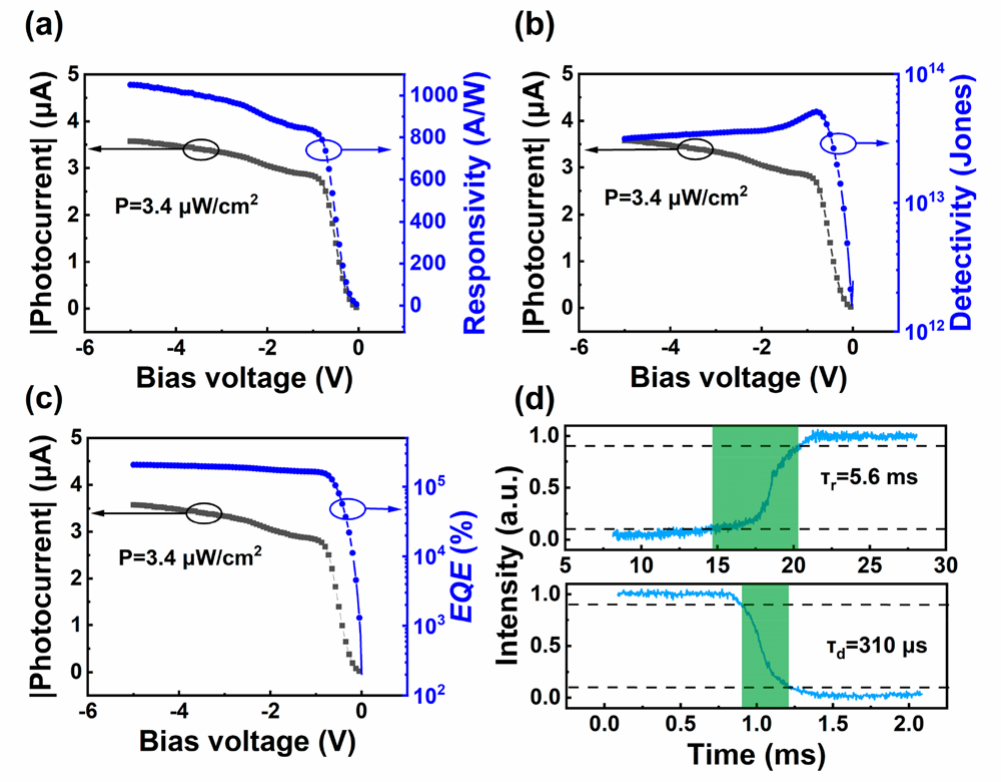
Photocurrent, responsivity (a), detectivity (b) and EQE (c) as a function of bias voltages. (d) Rise and decay time extracted from the dynamic current response within a single light pulse.

To understand the physical mechanism underlying the enhancement of photoresponse performance in the GNR/Si heterojunction with an interfacial Al_2_O_3_ layer, energy band diagrams of the GNR/Si device and GNR/Al_2_O_3_/Si device are illustrated in Figure 5a and b, respectively. Theoretically, in the GNR/Si device, the photogenerated carriers are separated by the reverse electric-field, with the photogenerated holes flowing to the GNRs side and the photogenerated electrons flowing to the Si side, thereby generating the photocurrent. However, in the practical GNR/Si device, the nondense surface of the GNR film and the residual high-conductivity SWCNTs lead to a large amount of reverse current leakage paths, which results in high dark current. The weak photogenerated current compared to the dark current is hardly to be observed. In contrast, the interfacial Al_2_O_3_ layer in the GNR/Al_2_O_3_/Si device provides a Schottky barrier and effectively blocks the leakage current paths but allows the effect hole tunneling transportation that contributed to the photo current.^33−35^ When the Al_2_O_3_ barrier layer was inserted between the Si and GNR film, the dark current was reduced because the holes must tunnel through the barrier to reach the GNR from Si under a reverse bias. The barrier causes hole accumulation at the interface. The lower reverse current with the Al_2_O_3_ barrier would also result in a lower hole density in the GNR valence band. On the other hand, very little change in the electron distribution in the device is expected. This is because there are nearly no electrons in the p-type GNR. And therefore, no electron current is expected to flow from the GNR to Si under the reverse bias. As a result, the dark current of the GNR/Al_2_O_3_/Si device decreases by over 2 orders of magnitude compared to that of the GNR/Si device. Meanwhile, the GNR has an opening bandgap, it is also involved in absorption of photons, which leads to a higher photodetection performance.

**Figure 5:**
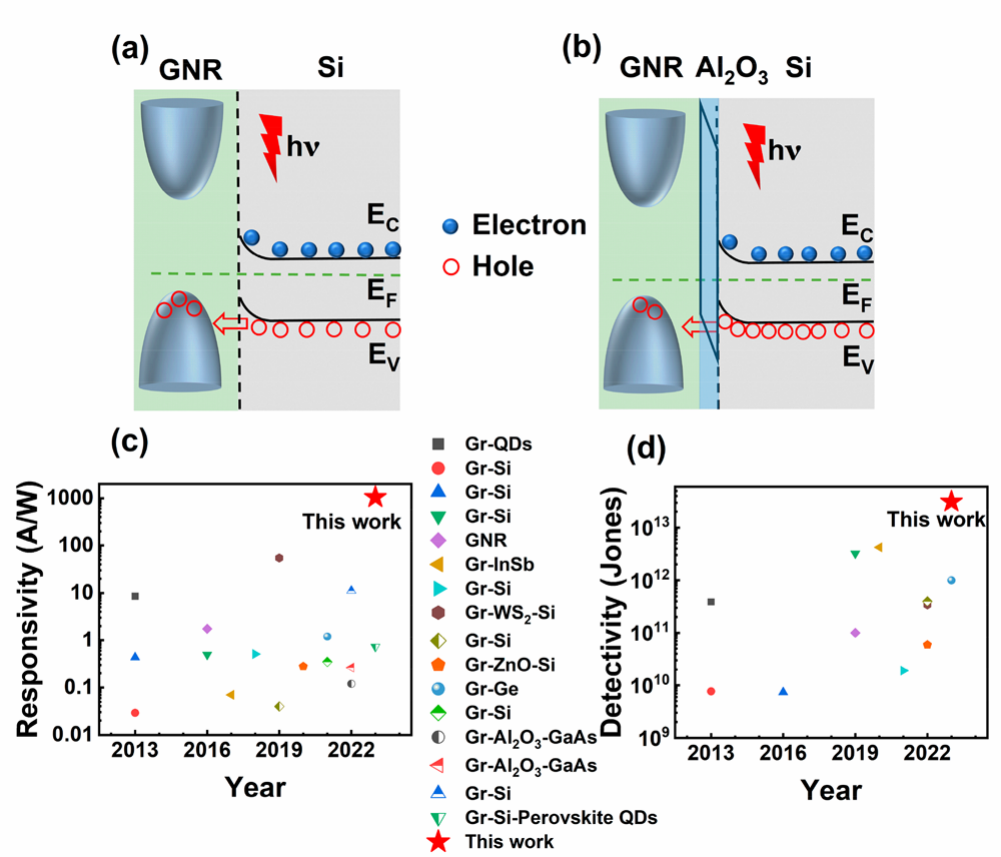
Energy band diagram of GNRs/Si without (a) and with (b) an Al_2_O_3_ interfacial layer under illumination. Comparison of responsivity (c) and detectivity (d) in this work with other reported graphene-based heterojunction photodetectors (Gr: graphene, QDs: quantum dots.).

To intuitively demonstrate the property level of the GNR/Al_2_O_3_/Si photodetector, the comparison of the device responsivity and detectivity with recently reported works are shown in Figure 5c and 5d, respectively. In this work, graphene nanoribbons have an opening bandgap of up to 1.81 eV, greatly enhancing their light absorption. In general, the responsivity of a photodetector is positively correlated with the mobility of absorbing material,^36−38^ and GNR maintains a high mobility of over 840 cm^2^/(V·s).^22^ These two factors are mainly responsible for the high photoresponsivity. To the best of our knowledge, the responsivity of the GNR/Al_2_O_3_/Si photodetector is the highest value for the reported graphene-based heterojunction photodetectors to date.^35,39−48^ As can also be seen in Figure 5d, the detectivity of the device is also in the leading position. In addition, the device demonstrates a fast response time, and the photocurrent also possesses high linearity with light power density, suggesting a high potential for practical applications.

In conclusion, the p-type GNR/n-type Si heterojunction with an interfacial Al_2_O_3_ layer is fabricated as a photodetector, demonstrating great optoelectrical properties under laser illumination with a wavelength of 635 nm. The dark current of the photodetector is reduced by over 2 orders of magnitude under the bias voltage of −5 V by introducing the interfacial Al_2_O_3_ layer. Due to the significant reduction of the dark current, the photodetector exhibits an excellent response to light illumination. At a bias voltage of −5 V, the photodetector exhibits an ultrahigh responsivity of 1052 A/W, surpassing the previous graphene-based heterojunction photodetector by several orders of magnitude. A detectivity of 3.1 × 10^13^ Jones, an EQE of over 10^5^ % and a response time of 310 μs are observed, which are also among the best values for reported graphene-based heterojunction photodetectors. The GNR-based photodetector also possesses a high responsivity and detectivity of 1.04 A/W and 2.45 × 10^12^ Jones even under 0 V bias, indicating a potential for self-powered photodetectors. These results demonstrate that the GNR-based photodetectors are promising candidates for future high-performance and low-cost photodetectors.

## Supporting Information Available

Materials and methods, introduction of GNRs, AFM images and height distributions of a SWCNT and GNR, current–voltage and current–time measurements of GNR/2.5 nm-Al_2_O_3_/Si, GNR/5 nm-Al_2_O_3_/Si and Al_2_O_3_/Si devices, current−voltage and current–time measurements of Au-Al_2_O_3_/Si device without GNR layer, dark currents of the GNR/Al_2_O_3_/Si device measured at a bias of 0 and −5 V, light absorption spectra of planar Si, GNR film and GNR/Al_2_O_3_/Si heterostructure, dynamic photocurrent response measured within a long time (PDF)
