# Supplementary material for: High-Performance Photodetectors Based on Semiconducting Graphene Nanoribbons
Source: Nano Lett. 2023 Nov 27;24(1):165–71. doi: 10.1021/acs.nanolett.3c03563 (PMC10786164; doi:10.1021/acs.nanolett.3c03563)
Supplement: Supplementary file 1 — nl3c03563_si_001.pdf [file nl3c03563_si_001.pdf]

Supporting Information for

High-performance Photodetectors Based on Semiconducting Graphene Nanoribbons

Mingyang Wang[a], Xiaoxiao Zheng[a], Xiaoling Ye[a], Wencheng Liu[a], Baoqing Zhang[a], Zihao Zhang[a], Rongli Zhai[a], Yafei Ning[a, b], Hu Li[*, a, b] and Aimin Song[*, a, c]

[a] Shandong Technology Centre of Nanodevices and Integration, School of Microelectronics, Shandong University, Jinan 250101, China.

[b] Shenzhen Research Institute of Shandong University, Shenzhen, 518063, China.

[c] Department of Electrical and Electronic Engineering, University of Manchester, M13 9PL, Manchester, U.K.

*Email: Hu.Li@sdu.edu.cn; A.Song@mancehster.ac.uk

Supplementary Text

Materials and Methods

Fabrication of GNRs

50 mg of single-walled carbon nanotubes (SWCNTs, Sigma Aldrich) were first annealed at 300 ℃ for 30 minutes under air condition and then immersed in 50 mL concentrated sulfuric acid (Sinopharm) for 2 hours. Next, 25 mg of $KMnO_4$ (Sigma Aldrich) was added into the mixture and then stirred for 30 minutes at 45 ℃ until $KMnO_4$ was fully consumed. The solution mixture was then poured into 200 mL of ice deionized water, then the mixture was filtered by a 0.45 μm PTFE filtration to separate reacted SWCNTs. The reacted SWCNTs were washed by a copious water and then dried in air. To obtain the GNR solution, 20 mg of the as-fabricated reacted SWCNTs was dispersed in 200 mL of 1% aq sodium dodecyl benzene sulfonate (Sigma Aldrich) solution and then sonicated for 60 minutes.

Fabrication of the GNR/Al_2O_3/Si heterojunctions

The process flow of GNR-based heterojunction photodetectors is presented in Figure 1(a). First, the n-type Si wafers were cleaned with acetone, ethanol, water and dried by N_2. Then the Si wafers were immersed in a 1% HF solution for 10s to eliminate the thin self-oxidizing layer on the surface. A 10 nm-thick Al_2O_3 film was deposited on the cleaned Si wafer by an atomic layer deposition (ALD). Then a 10 μL GNR aqueous solution was drop-casted on the surface of Al_2O_3 and dried for overnight. Prior to the deposition of electrodes, the sample was annealed in Ar atmosphere at 400 ℃ for 2 hours. Then 50 nm Au were

deposited as the top electrodes on the surface of the fabricated GNR film. The active area of the device is 0.1 mm^2.

Characterization of GNRs and the fabricated photodetectors

Raman spectra and photoluminescence spectra of SWCNTs and GNRs are characterized by the Raman Spectrometer (RENISHAW, invia) with a special excitation wavelength of 523 nm. An atomic force microscope (AFM, Benyuan CSPM5500) is used to characterize the morphology of SWCNTs and GNRs. The cross-section image is characterized by a transmission electron microscope (TEM, FEI Talos F200X). The current-voltage characteristics of devices is measured by an Agilent 2902A under air and dark condition. The optoelectrical performance of devices is measured under a laser illumination with a wavelength of 635 nm. The laser power density is obtained by an optical power meter (Ophir Nova II). An oscilloscope (Keysight MSOX6004A) is used to evaluate the response speed of the photodetector.

Effect of Al_2O_3 thickness gradient on device performance

We have investigated the performance of devices with different thickness of Al_2O_3 layers. Figure S2 shows device performance with Al_2O_3 thickness of 2.5 nm and 5 nm. The reverse saturation currents of the devices gradually decrease with increasing Al_2O_3 thickness, from 0.8 mA of the device with 0 nm Al_2O_3 to 3.6 μA of the device with 10 nm Al_2O_3. The devices with 2.5 nm and 5 nm Al_2O_3 exhibit reverse currents of 21.7 μA and 18.9 μA, which are lower than those of the device without the Al_2O_3 layer. In addition, in order to determine the best Al_2O_3 thickness, we also investigated the photodetection performance

of devices with different Al_2O_3 thickness. As shown in Fig. S2 and Fig. 2, the 10 nm Al_2O_3-device exhibits higher photocurrent and lowest dark current in the reverse bias region under the same power of light stimulation. The current response show that at 0 V bias, the 10nm Al_2O_3-device shows high and stable photocurrent. Under -5 V bias, the 10 nm Al_2O_3-device also shows higher photocurrent and lower dark current.

Background introduction of GNRs

Graphene has experienced great progress in recent years, owing to its myriad of advantages, including ultra-high carrier mobility and exceptional mechanical properties and so on[1-3]. However, graphene develops slowly in the field of photoelectric devices, especially when graphene is used as the light absorption medium. This is primarily attributable to graphene's intrinsic zero-bandgap characteristic, which results in a low optical gain[4]. Hence, graphene nanoribbons (GNRs) attract much attention due to their opening bandgap which induced by strong lateral quantum confinement[5]. Previous work predicted that GNRs are direct bandgap semiconductors[6-8], which gives it unlimited potential to replace graphene as a light absorption medium for fabricating high-performance photodetectors. However, theoretical research shows that the width of graphene nanoribbons must be less than 3 nm to produce a considerable band gap (>0.7 eV) to produce a sufficiently strong quantum confinement effect[9]. Therefore, it is very difficult to produce such a narrow GNR with high-precision lithography technology. The method of obtaining GNR by unzipping SWCNTs stands out among many methods of synthesizing GNR because of its solution synthesis and no need of vacuum. Meanwhile, the solution process gives the potential to prepare large-area GNR

films, which is very important, because it is difficult for traditional two-dimensional materials to fabricate large-area films.

Supplementary Figures

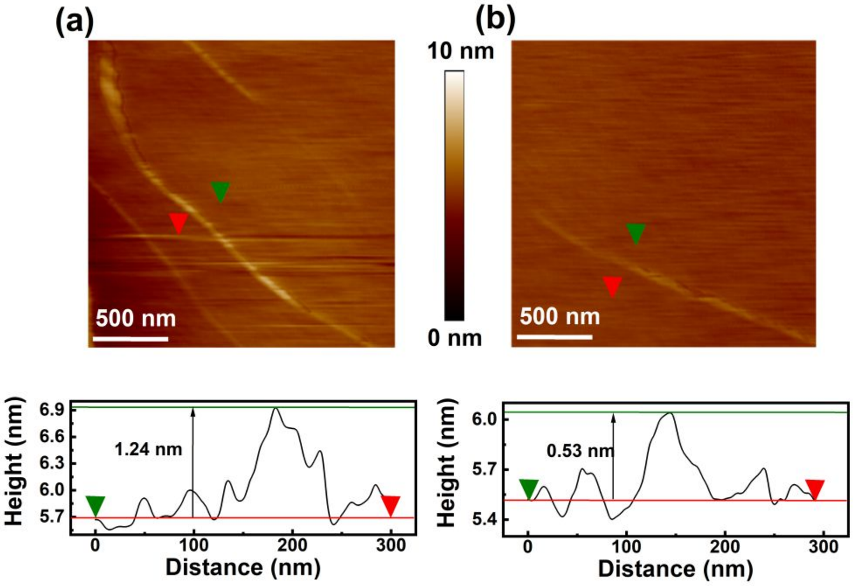

Figure S1. AFM images and height distributions of a SWCNT(a) and GNR (b).

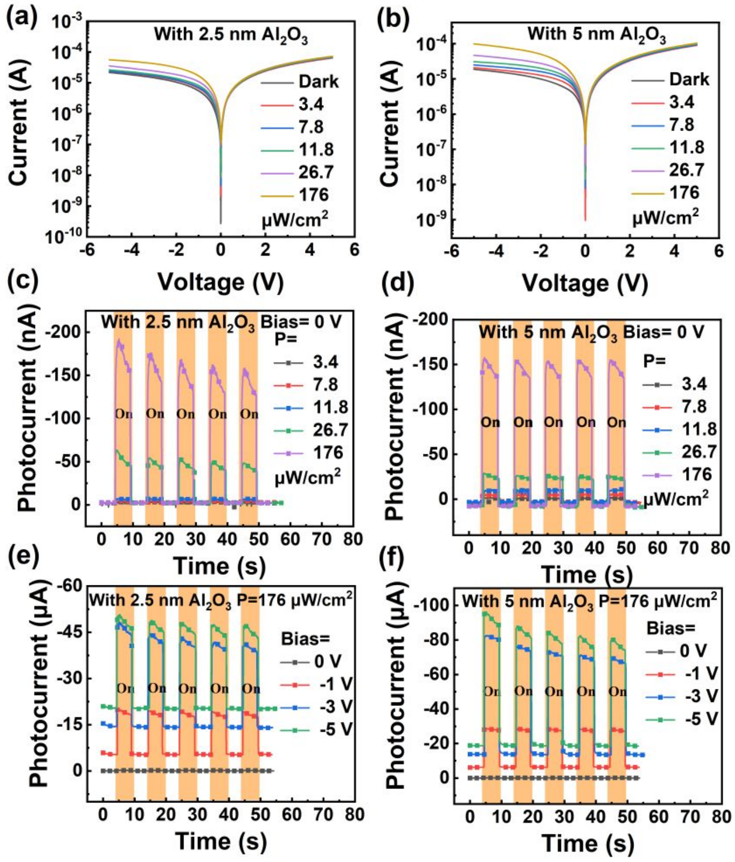

Figure S2. Current-voltage (I-V) curves of GNR/Si photodetectors with 2.5 (a) and 5 nm-

thick(b) Al_2O_3 interfacial layers measured under 635 nm laser irradiation at a light power density varying from 3.4 to 176 μW/cm^2. Dynamic current response of GNR/Si photodetectors with 2.5(c), 5 nm-thick(d) Al_2O_3 interfacial layers at 0 V bias. Dynamic current response of GNR/Si photodetectors with 2.5(c), 5 nm-thick(d) Al_2O_3 interfacial layers at -5 V bias.

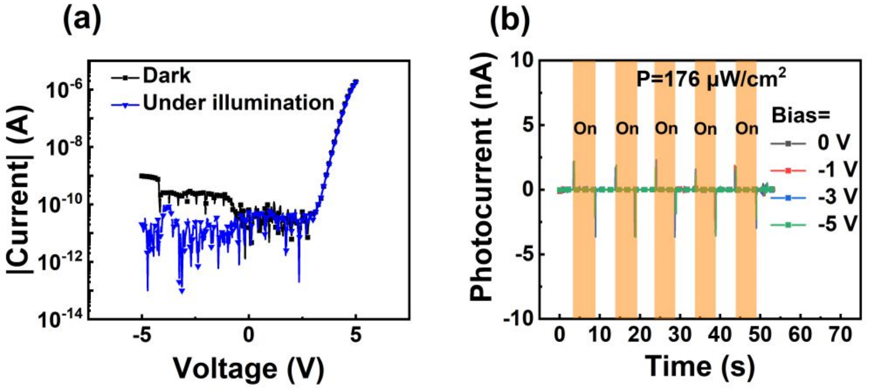

Figure S3. (a) Current-voltage (I-V) curves of Au-Al_2O_3-Si device without GNR layer measured under a laser illumination with a light power density of 176 μW/cm^2 and dark condition. Dynamic current response of Au-Al_2O_3-Si device without GNR layer at different bias.

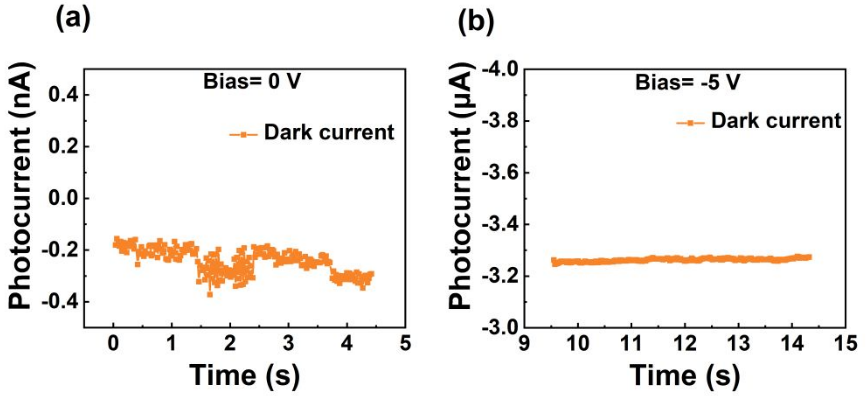

Figure S4. Dark currents of the GNR/Al_2O_3/Si device measured at the bias of (a) 0 V and (b) -5 V.

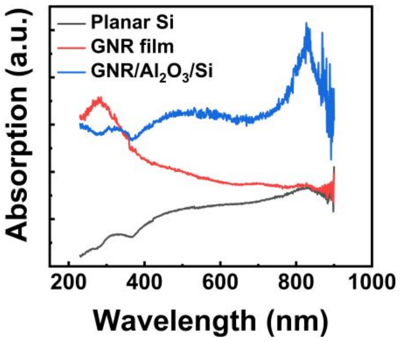

Figure S5. Light absorption spectra of planar Si, GNR film and GNR/Al_2O_3/Si heterostructure.

As shown in Figure S5, the absorption intensity of the planar Si and GNR film is both lower than that of the GNR/Al_2O_3/Si heterostructure. This can be attributed to the enhancement of the photogenerated carrier separation efficiency by the built-in electric field in the heterojunction leading to an increase in the apparent absorption intensity.

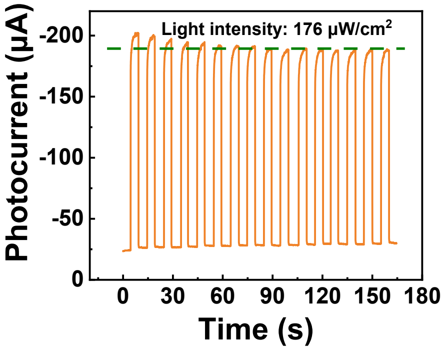

Figure S6. Dynamic photocurrent response measured within a long time.

<u>Reference:</u>

(1) Kim, K. S.; Zhao, Y.; Jang, H.; Lee, S. Y.; Kim, J. M.; Kim, K. S.; Ahn, J. H.; Kim, P.; Choi, J. Y.; Hong, B. H., Large-scale pattern growth of graphene films for stretchable

transparent electrodes. Nature **2009**, 457, 706-710.

(2) Bonaccorso, F.; Colombo, L.; Yu, G.; Stoller, M.; Tozzini, V.; Ferrari, A. C.; Ruoff, R. S.; Pellegrini, V., 2D materials. Graphene, related two-dimensional crystals, and hybrid systems for energy conversion and storage. Science **2015**, 347, 1246501.

(3) Mehdi Pour, M.; Lashkov, A.; Radocea, A.; Liu, X.; Sun, T.; Lipatov, A.; Korlacki, R. A.; Shekhirev, M.; Aluru, N. R.; Lyding, J. W.; Sysoev, V.; Sinitskii, A., Laterally extended atomically precise graphene nanoribbons with improved electrical conductivity for efficient gas sensing. Nat. Commun. **2017**, 8, 820.

(4) Zhang, B. Y.; Liu, T.; Meng, B.; Li, X.; Liang, G.; Hu, X.; Wang, Q. J., Broadband high photoresponse from pure monolayer graphene photodetector. Nat. Commun. **2013**, 4, 1811.

(5) Chong, M. C.; Afshar-Imani, N.; Scheurer, F.; Cardoso, C.; Ferretti, A.; Prezzi, D.; Schull, G. Bright Electroluminescence from Single Graphene Nanoribbon Junctions. Nano Lett. **2018**, 18, 175−181.

(6) Senkovskiy, B. V.; Pfeiffer, M.; Alavi, S. K.; Bliesener, A.; Zhu, J.; Michel, S.; Fedorov, A. V.; German, R.; Hertel, D.; Haberer, D.;Petaccia, L.; Fischer, F. R.; Meerholz, K.; van Loosdrecht, P. H. M.;Lindfors, K.; Grüneis, A. Making Graphene Nanoribbons Photoluminescent. Nano Lett. **2017**, 17, 4029−4037.

(7) Ma, F.; Guo, Z.; Xu, K.; Chu, P. K. First-Principle Study of Energy Band Structure of Armchair Graphene Nanoribbons. Solid State Commun. **2012**, 152, 1089−1093.

(8) Yang, L.; Park, C.-H.; Son, Y.-W.; Cohen, M. L.; Louie, S. G. Quasiparticle Energies

and Band Gaps in Graphene Nanoribbons. Phys. Rev. Lett. **2007**, 99, 186801.

(9) Poljak, M.; Wang, K. L.; Suligoj, T. Variability of Bandgap and Carrier Mobility Caused by Edge Defects in Ultra-Narrow Graphene Nanoribbons. Solid-State Electron. 2015, 108, 6 7−74.
